# PD216 Efficiency Frontier Analysis: Supporting Sustainable Non-Small Cell Lung Cancer Healthcare Policies In Brazil

**DOI:** 10.1017/S0266462324004379

**Published:** 2025-01-07

**Authors:** Marcus Carvalho Borin, Carina Rejane Martins, Daniel Pitchon dos Reis, Geraldo Jose Coelho Ribeiro, Julia Teixeira Tupinambas, Karina de Castro Zocrato, Lelia Maria de Almeida Carvalho, Marcela Pinto de Freitas, Maria da Gloria Cruvinel Horta, Mariana Michel Barbosa, Mariza Cristina Torres Talim, Sergio Adriano Loureiro Bersan, Silvana Marcia Bruschi Kelles

## Abstract

**Introduction:**

Informed healthcare policies in Brazil rely on robust health technology assessment (HTA), especially for conditions like non-small cell lung cancer (NSCLC). We present an efficiency frontier analysis to evaluate NSCLC treatments that correlates annual treatment costs with clinical outcomes, offering a systematic approach to enhance decision-making in the Brazilian healthcare context.

**Methods:**

This quantitative study analyzed NSCLC drug costs within the Brazilian healthcare system and the clinical efficacy data of pivotal studies. The data were analyzed using Python and R software. The dataset comprised drug costs and hazard ratios for overall survival. After data preparation, which involved normalization and outlier management, we constructed an efficiency frontier by ranking drugs based on cost and effectiveness. A linear regression model was then developed to extrapolate this frontier, deriving a formula that predicts treatment costs for specified improvements in overall survival.

**Results:**

The analysis delineated an efficiency frontier and revealed cost-effective NSCLC treatments in Brazil. The following linear regression equation was derived: overall survival = (1.033551 − 0.000003) × treatment cost (USD). This allows for the estimation of appropriate treatment costs for new therapies based on their expected clinical outcomes. This initial model provides a foundation for estimating the economic impact of new treatments.

**Conclusions:**

This preliminary efficiency frontier analysis offers a novel perspective for evaluating NSCLC treatment strategies in Brazil to support sustainable healthcare policy decisions. The model is subject to limitations due to the absence of a systematic literature review. However, it represents an initial step towards a more comprehensive HTA framework. Further research should refine the model by including systematic data collection and analysis.

